# Subjective Vividness of Kinesthetic Motor Imagery Is Associated With the Similarity in Magnitude of Sensorimotor Event-Related Desynchronization Between Motor Execution and Motor Imagery

**DOI:** 10.3389/fnhum.2018.00295

**Published:** 2018-07-31

**Authors:** Hisato Toriyama, Junichi Ushiba, Junichi Ushiyama

**Affiliations:** ^1^Graduate School of Media and Governance, Keio University, Fujisawa, Japan; ^2^Department of Biosciences and Informatics, Faculty of Science and Technology, Keio University, Yokohama, Japan; ^3^Keio Institute of Pure and Applied Sciences, Yokohama, Japan; ^4^Faculty of Environment and Information Studies, Keio University, Fujisawa, Japan; ^5^Department of Rehabilitation Medicine, Keio University School of Medicine, Keio University, Tokyo, Japan

**Keywords:** motor imagery, electroencephalogram, sensorimotor rhythm, corticospinal excitability, the Kinesthetic and Visual Imagery Questionnaire

## Abstract

In the field of psychology, it has been well established that there are two types of motor imagery such as kinesthetic motor imagery (KMI) and visual motor imagery (VMI), and the subjective evaluation for vividness of motor imagery each differs across individuals. This study aimed to examine how the motor imagery ability assessed by the psychological scores is associated with the physiological measure using electroencephalogram (EEG) sensorimotor rhythm during KMI task. First, 20 healthy young individuals evaluated subjectively how vividly they can perform each of KMI and VMI by using the Kinesthetic and Visual Imagery Questionnaire (KVIQ). We assessed their motor imagery abilities by summing each of KMI and VMI scores in KVIQ (KMI_total_ and VMI_total_). Second, in physiological experiments, they repeated two strengths (10 and 40% of maximal effort) of isometric voluntary wrist-dorsiflexion. Right after each contraction, they also performed its KMI. The scalp EEGs over the sensorimotor cortex were recorded during the tasks. The EEG power is known to decrease in the alpha-and-beta band (7–35 Hz) from resting state to performing state of voluntary contraction (VC) or motor imagery. This phenomenon is referred to as event-related desynchronization (ERD). For each strength of the tasks, we calculated the maximal peak of ERD during VC, and that during its KMI, and measured the degree of similarity (ERD_sim_) between them. The results showed significant negative correlations between KMI_total_ and ERD_sim_ for both strengths (*p* < 0.05) (i.e., the higher the KMI_total_, the smaller the ERD_sim_). These findings suggest that in healthy individuals with higher motor imagery ability from a first-person perspective, KMI efficiently engages the shared cortical circuits corresponding with motor execution, including the sensorimotor cortex, with high compliance.

## Introduction

Motor imagery has been defined as a dynamic internal representation of a given motor act without any overt motor output (Decety and Grezes, 1999), and suggested to utilize common neural substrate with motor execution (Decety, 1996; Jeannerod and Frak, 1999; Gerardin et al., 2000; Hanakawa et al., 2003). By comparing different conditions such as stroke and lesions, neural functions of motor imagery have been clarified to date. For example, the roles of ventral and dorsal premotor cortices perform planning, preparation, execution, and imagery of motor acts (Hetu et al., 2013). Posterior parietal, cerebellar, and premotor regions, which are known to contain internal representations for both movements and the body itself, are thought to play a decisive role in the generation of motor imagery (Lorey et al., 2011). The superior fronto-occipital fasciculus of the long associative fibers are related to the generation of motor imagery, which mediate the integration of visual and sensory information for motor planning and control (Oostra et al., 2016).

In the field of psychology, the ability of motor imagery has been often measured via introspective reports of the vividness of imagery experiences through validated questionnaires such as the Kinesthetic and Visual Imagery Questionnaire (KVIQ) (Malouin et al., 2007). Two ways of motor imagery, the kinesthetic motor imagery (KMI) and the visual motor imagery (VMI), has been discussed. KMI requires us to feel a movement from a first-person perspective, while the VMI requires us to self-visualize a movement from first and third-person perspectives (Hall et al., 1985; Stinear et al., 2006; Malouin et al., 2007; Guillot et al., 2009). Several psychological studies have reported longitudinal changes in motor imagery ability due to aging (Malouin et al., 2010) or motor imagery training (Oostra et al., 2015) by measuring KMI and VMI scores of the introspective questionnaire.

In the field of physiology, it is well known that the electroencephalogram (EEG) sensorimotor rhythm changes from the resting state to the motor execution or motor imagery state. This phenomenon reflects a decrease in the power of EEG over the primary sensorimotor area in the alpha (7–14 Hz) and beta (15–35 Hz) bands indicating underlying cortical cells to be desynchronized (Pfurtscheller, 1981, 1992; Pfurtscheller and Berghold, 1989; Nunez and Srinivasan, 2006). Thus, this phenomenon is called “event-related desynchronization (ERD).” Recently, several studies have provided evidences suggesting that ERD reflects increased neuronal excitability in the corticospinal system, by combining EEG recordings with transcranial magnetic stimulation (TMS) (Hashimoto and Rothwell, 1999; Hummel et al., 2002; Takemi et al., 2013), functional magnetic resonance imaging (fMRI) (Formaggio et al., 2008, 2010; Yuan et al., 2010; Mokienko et al., 2013), and F-wave assessment (Takemi et al., 2015).

In brain–computer interface (BCI) studies based on EEG, persons with low and high BCI aptitude prefer different forms of motor imagery (i.e., persons with high KMI score produced high BCI performance) (Vuckovic and Osuagwu, 2013; Marchesotti et al., 2016). In addition, correlation between BCI accuracy and ERD magnitude was reported (Kaplan et al., 2016). It is also known that BCI performance is correlated with sensorimotor predictor (Blankertz et al., 2010). Based on these findings, it is likely that persons with high KMI score produce large ERD magnitude around the sensorimotor area. Nevertheless, no correlation between them was observed (Vasilyev et al., 2017). To begin with, the procedure of measuring motor imagery in most of psychological questionnaires is as follows. In one trial, participants perform motor execution and then perform motor imagery. This is one trial and they perform motor execution and imagery with different movements for each trial. In other words, they performed motor imagery by referring the preceding motor execution. Additionally, motor imagery utilizes common neural substrate with motor execution (Decety, 1996; Jeannerod and Frak, 1999; Gerardin et al., 2000; Hanakawa et al., 2003). Therefore, it is hypothesized that the degree of similarity in EEG sensorimotor rhythm between motor execution and motor imagery is correlated with the motor imagery ability, especially during KMI.

To test this hypothesis, we first evaluated the subjective vividness of KMI and VMI by KVIQ for each participant. Next, according to the procedure of KVIQ, each participant repeated voluntary wrist dorsiflexion and its KMI, and the magnitudes of ERD during the two tasks and their similarity were evaluated. Finally, we examined the association between the psychological motor imagery ability and physiological EEG sensorimotor rhythm.

## Materials and Methods

### Ethical Approval

This study was conducted in accordance with the Declaration of Helsinki amended by the 64th WMA General Assembly, Fortaleza, Brazil, October 2013. All experimental protocols and procedures were approved by the Research Ethics Committee in Shonan Fujisawa Campus, Keio University (Approval Number 167). After fully understanding a detailed explanation of the purpose, experimental procedures, potential benefits and risks, which were provided from the examiners, all participants documented informed consent before participation to this experiment.

### Participants

Twenty healthy individuals (12 males, 8 females, aged 18–25 years) participated in the study. They were either undergraduates or graduates of universities. All were right-handed without any medical and/or physiological disorders.

### Psychological Assessments

#### Procedures

A well-established questionnaire was used for psychological assessment of motor imagery: The Kinesthetic and Visual Imagery Questionnaire (KVIQ-20) (Malouin et al., 2007). In the KVIQ, the participants sat in a comfortable chair. Initially, the participants made a simple movement such as elbow flexion/extension, forward trunk flexion, and knee extension. Next, the participants performed KMI or VMI of the preceding movement according to the examiner’s instruction and then assessed the vividness of the preceding motor imagery on a 5-point ordinal scale (i.e., the greater the scale, the more vivid the motor imagery). They repeated this process for 10 different movements from two dimensions of motor imagery.

#### Analyses

We evaluated the vividness of motor imagery by summing all KVIQ scores for KMI (KMI_total_) and VMI (VMI_total_), respectively. In addition, because the participants engaged in physiological experiment by using their upper limb, we evaluated the vividness of motor imagery just for upper limb movements, by summing the KVIQ scores of upper limb items for KMI (KMI_upper_) and VMI (VMI_upper_), respectively.

### Physiological Assessments

#### Recordings

**FIGURE 1:**
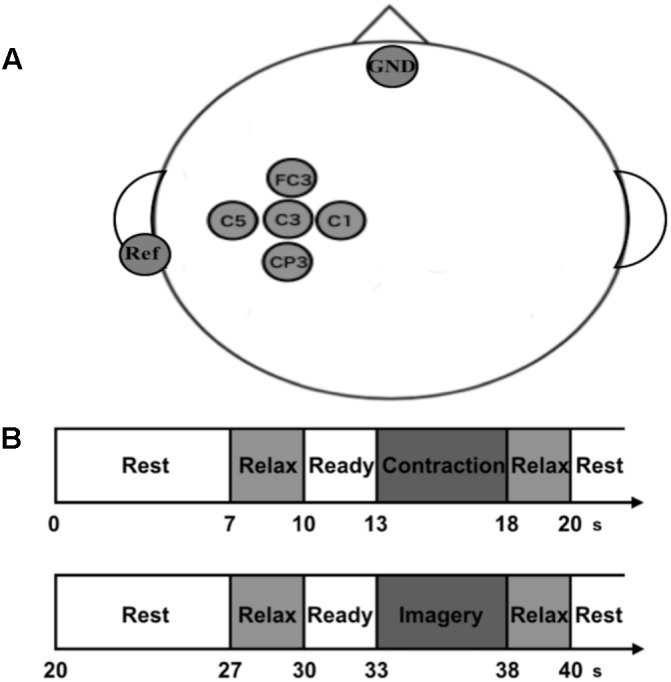
Experimental recordings, paradigm, and setup. **(A)** Electroencephalogram (EEG) channel locations used in the present study. We recorded EEG signals from five electrodes on the scalp over the sensorimotor area of right forearm. The ground electrode (GND) was placed over the forehead, and the reference electrodes (Ref) were located at the left earlobe. **(B)** Experimental paradigm of the neurophysiological assessment. Participants repeated pairs of isometric voluntary wrist-dorsiflexion (VC) and its kinesthetic motor imagery (KMI).

Scalp EEG signals were recorded from five scalp positions (FC3, C5, C3, C1, and CP3) around the sensorimotor area related to the right upper limb arranged according to the international 10–20 system of electrode placement (**Figure 1A**). Five passive Ag/AgCl electrodes with a diameter of 18 mm were mounted on an electrode cap (g.GAMMAcap 1027; Guger Technologies, Graz, Austria). We also placed reference and ground electrodes on the right earlobe and forehead, respectively. Surface electromyogram (EMG) signals were recorded from the extensor carpi radialis muscle (ECR). Two passive Ag/AgCl electrodes with a diameter of 10 mm were placed over its muscle belly. The inter-electrode distance was 20 mm. All EEG and EMG signals were amplified and band-pass filtered (EEG, 0.5–200 Hz; EMG, 5–1000 Hz) using a linked biosignal recording system (g.BSamp 0201a; Guger Technologies, Graz, Austria). Force signals were recorded by a wrist dynamometer (TCF-50N; Takei Scientific Instruments Co., Ltd, Niigata, Japan), and amplified and low-pass filtered with a cut-off frequency of 100 Hz by using a force amplifier (DPM-711B; Kyowa Electronic Instruments Co., Ltd., Tokyo, Japan). All the analog EEG, EMG and force signals were converted to digital signals at a sample rate of 1000 Hz by an AD converter with 16-bit resolution (NI USB-6259 BNC, National Instruments, Austin, TX, United States) that was controlled by a data-logger software originally designed using MATLAB software (The MathWorks, Inc., Antic, MA, United States).

#### Procedures

After the psychological assessments using the KVIQ, the participants engaged in the physiological experiment. They sat in a comfortable chair with their right hand set perpendicular to the armrest, facing the palm inward. A 24-inch computer monitor was placed with a horizontal distance of 1 m in front of their eyes. The information of their exerted force and the required strength were given through the monitor. The monitor displayed instructions such as “Rest,” “Relax,” “Ready,” “Contraction,” and “Image.” It also displayed the exerted force by wrist-dorsiflexion as a red marker and the target force as a blue line. They were instructed to exert force so that the red marker could follow the blue line as accurately as possible during “Contraction” phase. During the other phases, they kept their hand still so that the red marker could stay on the baseline. We adopted wrist-dorsiflexion as the motor execution task because numerous neurophysiological or neurorehabilitation studies measuring ERD have used a hand movement as a subject of motor imagery (Cassim et al., 2000; Hummel et al., 2002, 2004; Müller et al., 2003; Shindo et al., 2011; Ang et al., 2014).

Before the experiment, they performed several practice trials of maximal voluntary contractions (MVCs) of wrist-dorsiflexion. After the practice, they performed the MVC task once. We calculated the peak force from this trial and adopted it as MVC force for each participant. We also checked that this MVC force was within the range of the practice trials. After we set the MVC force, the main experiment was performed. Each trial was started with the presentation of the word “Rest” at the top center of monitor. For “Rest” phases, they could do anything freely such as adjusting posture and blinking strongly. Seven seconds later, the word “Relax” was presented for 3 s. For this phase, they were required to get relaxed as they could without any movement. Then the word “Ready” was presented for 3 s with a short sound given every second. For this phase, they prepared for the next instruction. After the “Ready” phase, the monitor displayed the word “Contraction” and they performed isometric voluntary wrist-dorsiflexion by their right hand at 10 or 40% of MVC for 5 s. After “Relax” presentation for 2 s, the contraction task was finished, and the imagery task was begun. After the presentation of “Rest,” “Relax,” and “Ready” in the same manner, the word “Image” was displayed dimly and they performed KMI of the preceding contraction for 5 s with their eyes opened and without any movement. Note that they performed KMI with no online neurofeedback to uniform the procedure of the physiological experiment with the psychological KVIQ assessment. During KMI, we checked that no EMG activity was occurred. In the end, the word “Relax” was presented again for 2 s and the imagery task was finished. This is the flow for a trial (**Figure 1B**). We set two trials for each strength in a random order within a set and repeated this set for 15 times with intervals of 3–5 min. In total, they performed 30 trials including contraction and imagery tasks for both 10 and 40% of MVC, and it took 40 min. We set two strengths of contraction and its KMI to prevent the participants from habituating themselves to performing the same strength of KMI, and to purely evaluate changes in EEG sensorimotor rhythm when performing KMI by using the preceding movement as a reference.

#### Analyses

The ERD has been defined as a decrease in the EEG power in the alpha-and-beta band compared with a baseline period (Pfurtscheller, 1981; Pfurtscheller and Berghold, 1989). In this experiment, we calculated ERDs for four tasks such as 10% voluntary contraction (VC), 10% KMI, 40% VC, and 40% KMI, respectively, by referring a baseline period defined as the last 1 s in the “Relax” phase. First, the EEG signal from C3 was subtracted by the average of four-nearest neighboring electrodes (FC3, C5, C1, and CP3). This derivation method is referred to as a small Laplacian and is known to highly emphasize cortical activity originating below the electrode of interest (McFarland et al., 1997). If the amplitude of Laplacian-derived EEG exceeded 50 μV, we judged this trial to have an artifact. These trials were excluded for further analyses. For each task, we made a dimensional matrix of the Laplacian-derived EEG data [20000 data points (= 20 s × 1000 Hz) × 30 trials]. Next, we extracted the thirty 1-s data windows in the same period from data for each trial and gathered them to provide 30-s of EEG data. Then we performed the fast Fourier transformation by Welch’s method for the 30-s data (window length, 1 s; window function, hanning-window; overlap, 0) to compute the power spectral densities (PSDs) of EEG signals. We repeated this process by sliding the 1-s data windows in 50 ms steps, and as a result, obtained a two dimensional matrix showing the time dependent changes in EEG PSD for each task (time × frequency). The ERD was calculated with a time resolution of 50 ms and a frequency resolution of 1 Hz, according to the following equation:

(1) $$\text{ERD}(f,t)=\frac{R(f)-A(f,t)}{R(f)}$$

where *A* is the EEG PSD at time *t*, frequency *f* and *R* is the mean PSD of the baseline period (the last 1-s in the “Relax” phase). This equation expresses a large ERD as a large positive value. The ERD is typically found over the primary sensorimotor cortex contralateral to the contracting or imaging limb, but the most reactive frequency band displaying ERD was slightly different across participants (Pfurtscheller and Neuper, 2006; Takemi et al., 2013). As for each participant, the 3 Hz of frequency-band showing the largest ERD was determined within alpha and beta bands (7–35 Hz) by using their EEG signals recorded in contraction tasks for each strength. It is known that an alpha band is the main component of sensorimotor rhythm and desynchronized during KMI (Pfurtscheller et al., 2006; Nagamori and Tanaka, 2016). As shown in the recent paper with EEG–fMRI simultaneous recording (Tsuchimoto et al., 2017), EEG beta component recorded at C3 is derived dominantly from the primary motor cortex, but partially from the primary somatosensory cortex. Former EEG study by Pfurtscheller (Pfurtscheller and Lopes da Silva, 1999) also shows the location of EEG beta component is slightly anterior to EEG alpha component but is partially overlapped. From these findings, EEG beta component at C3 in this study would be sensorimotor origin as well as EEG alpha component. By using averaged ERD data for the examined most reactive frequency band, the magnitude of ERD was measured by calculating the peak value of ERD for both contraction (ERD_V C_) and its KMI (ERD_KMI_) tasks (Takemi et al., 2015). As reported previously, the most reactive frequency bands slightly differ for each day and for each participant (Aftanas et al., 2002; Muller-Putz et al., 2005; Neuper et al., 2006; Hashimoto et al., 2010). Therefore, we found the most reactive frequency by shifting 3 Hz of frequency-band for each task (10 or 40% MVC) (**Table 1**).

**Table 1:** Data representing the scores of KVIQ, the frequency of interest, ERD magnitude during motor execution and motor imagery.

Participant	KVIQ scores				Frequency of interest (Hz)		ERD_V C_ (%)		ERD_KMI_ (%)	
	KMI_total_	VMI_total_	KMI_upper_	VMI_upper_	10%	40%	10%	40%	10%	40%
A	63	64	15	14	14–16	15–17	80.32	90.82	77.97	70.63
B	57	59	15	14	32–34	11–13	38.62	57.43	20.80	46.15
C	85	85	20	20	8–10	19–21	91.72	88.41	68.24	68.47
D	74	76	15	18	11–13	17–19	39.16	47.23	45.78	45.04
E	84	83	20	20	21–23	22–24	52.18	49.02	60.56	61.35
F	48	47	8	10	12–14	20–22	58.76	60.55	48.84	61.44
G	41	48	8	10	13–15	12–14	79.49	88.21	47.32	27.34
H	68	62	18	17	20–22	13–15	57.12	57.61	64.71	37.18
I	62	66	15	17	23–25	29–31	84.35	78.88	88.88	53.16
J	58	71	17	20	18–20	15–27	85.71	88.25	73.41	77.26
K	33	81	8	18	11–13	24–26	84.56	90.01	31.59	31.35
L	65	70	13	17	14–16	11–13	59.16	51.29	37.53	32.96
M	50	64	16	17	9–11	11–13	82.20	78.48	51.38	42.46
N	68	81	15	20	15–17	13–15	68.24	61.44	45.67	43.90
O	79	53	19	14	16–18	11–13	80.14	87.33	74.79	88.47
P	60	50	12	12	15–17	22–24	73.92	75.89	54.60	63.28
Q	69	74	14	19	12–14	13–15	61.99	65.77	57.99	60.82
R	45	62	10	14	15–17	11–13	78.46	71.16	40.62	38.46
S	39	50	11	15	20–22	16–18	60.78	78.99	38.66	45.29
T	56	75	14	17	13–15	11–13	65.71	74.24	58.61	53.18

*The total score of KVIQ is on an 85-point scale and the upper score is on a 20-point scale.*

Additionally, we measured the degree of similarity between ERD_V C_ and ERD_KMI_ (ERD_sim_). We applied ERD_sim_ to this study to evaluate how similarly the EEG sensorimotor rhythm changed between contraction and its KMI tasks. The equation of ERD_sim_ is as follows:

(2) $${\text{ERD}}_{\text{sim}}\text{=}\frac{{\text{ERD}}_{\text{VC}}{\text{-ERD}}_{\text{KMI}}}{{\text{ERD}}_{\text{VC}}{\text{+ERD}}_{\text{KMI}}}$$

We measured the ERD_sim_ because in the KVIQ, we evaluated how vividly the participants could perform motor imagery of the preceding movement. We calculated ERD_sim_ between 10% VC and 10% KMI tasks, and between 40% VC and 40% KMI tasks, respectively.

### Statistical Analyses

First, we performed Pearson’s correlations analyses between psychological and neurophysiological measures. As psychological measures assessing vividness of motor imagery, we used the KVIQ scores such as KMI_total_, VMI_total_, KMI_upper_, and VMI_upper_. As neurophysiological measures reflecting EEG sensorimotor rhythm, ERD_KMI_ and ERD_sim_ for both strengths were used. As there were 16 items to compare, Bonferroni-corrected *p*-values of 0.003 (*p* = 0.05/16) were used to indicate the statistical significance. The degree of freedom was all 18. Additionally, in order to measure the effects of imagined contraction strength on EEG sensorimotor rhythm, we used two-way repeated measure ANOVA (2 tasks × 2 strengths). The *p*-values of 0.05 were used to indicate the statistical significance. All statistical analyses were performed using MATLAB software.

## Results

**Table 1** shows the information including each score of KVIQ, frequency band of interest, ERD_V C_, and ERD_KMI_ for each strength from all participants.

**FIGURE 2:**
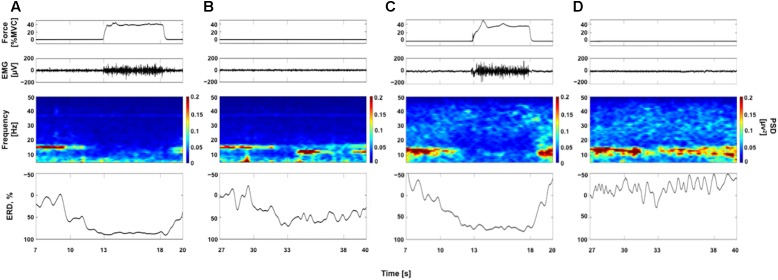
Examples of raw force and electromyogram (EMG) signals, EEG time frequency maps, and event-related desynchronization (ERD) curves. Panels **(A,B)** are from a single participant (Participant A, see **Table 1**) and panels **(C,D)** are from a single participant (Participant G, see **Table 1**). The raw force and EMG signals are from a trial and EEG time frequency map and ERD curve from average data of all trials. **(A,C)** Data for voluntary contraction at 40% of maximal effort (40% VC task). **(B,D)** Data for kinesthetic motor imagery of the 40% VC task (40% KMI task). See more detail in Supplementary Figure 1 showing ERD curves for all participants.

**Figure 2** represents typical examples of raw force and EMG signals, EEG time-frequency maps, and ERD curves obtained each from a participant (Participants A and G, see **Table 1**). **Figures 2A,C** show data obtained from 40% contraction task. In EEG time frequency map, we can clearly observe the decrease in the EEG power around 15 Hz in “Contraction” phase (13–18 s) compared with “Relax” phase (7–10 s). In **Figures 2B,D**, data obtained from 40% KMI task are shown. As seen in **Figure 2B**, the decrease in the EEG power was confirmed in “Image” phase (33–38 s) compared with “Relax” phase (27–30 s). The ERDs were started during “Ready” phase (30–33 s) and reached to the maximal value after beginning of the tasks in accordance with previous reports (Pfurtscheller and Aranibar, 1979; Pfurtscheller and Berghold, 1989; Pfurtscheller and Lopes da Silva, 1999). By comparing the time frequency maps and ERD curves in **Figures 2B,D**, the decrease in the EEG power was confirmed in “Image” phase in **Figure 2B**, but it was difficult to confirm the remarkable decrease in EEG power in **Figure 2D**. We showed **Figure 2** to confirm samples of ERD_sim_ variations between individuals.

First, two-way repeated measurement ANOVA with task (actual contraction or imagined contraction) and strength (10 or 40%) as factors revealed an effect of task (*F*_1,57_ = 45.21, *p* < 0.001). However, an effect of strength (*F*_1,57_ = 0.033, *p* = 0.856) and a task × strength interaction (*F*_1,57_ = 0.922, *p* = 0.341) were not revealed by the two-way ANOVA.

Next, we examined the extent to which the ERD magnitude itself reflects the vividness of motor imagery. Although KMI_total_ showed a tendency to correlate negatively with ERD_KMI_ in 10% KMI task (*r* = 0.487, *p* = 0.030) and with ERD_KMI_ in 40% KMI task (*r* = 0.533, *p* = 0.016), their *p*-values did not meet the significant level in this study. In addition, VMI_total_ was not correlated with ERD_KMI_ in 10% KMI task (*r* = 0.083, *p* = 0.728) and with ERD_KMI_ in 40% KMI task (*r* = -0.012, *p* = 0.961). Although KMI_upper_ showed a tendency to correlate negatively with ERD_KMI_ in 10% KMI task (*r* = 0.537, *p* = 0.015) and with ERD_KMI_ in 40% KMI task (*r* = 0.531, *p* = 0.016), their *p*-values did not meet the significant level in this study as well as KMI_total_. Moreover, VMI_upper_ was not correlated with ERD_KMI_ in 10% KMI task (*r* = 0.191, *p* = 0.419) and with ERD_KMI_ in 40% KMI task (*r* = 0.084, *p* = 0.725).

**FIGURE 3:**
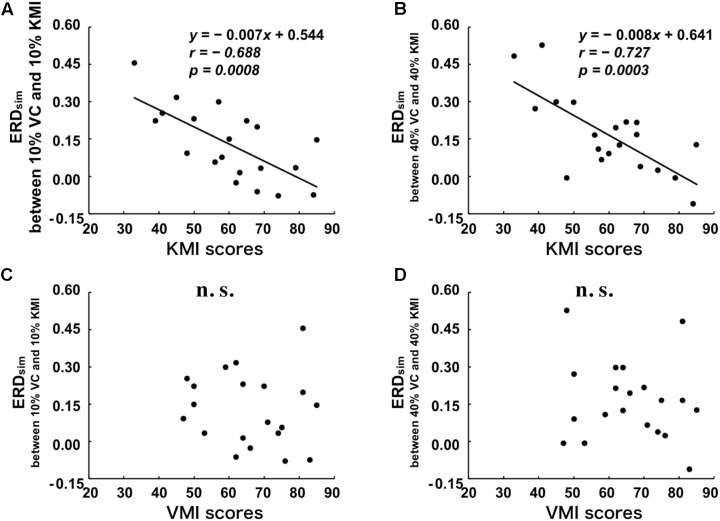
Relationships between the scores of the KVIQ and the degree of similarity (ERD_sim_) between VC and KMI tasks. **(A)** Relationship between KMI_total_ and ERD_sim_ between 10% VC and 10% KMI tasks. **(B)** Relationship between KMI_total_ and ERD_sim_ between 40% VC and 40% KMI tasks. **(C)** Relationship between VMI_total_ and ERD_sim_ between 10% VC and 10% KMI tasks. **(D)** Relationship between VMI_total_ and ERD_sim_ between 40% VC and 40% KMI tasks. Linear regression equations, Pearson’s correlation coefficients, *r*, and *p*-values are represented in each figure, if the relationship is significant. The black lines indicate the estimated regression lines.

Additionally, to examine the extent to which the similarity between ERD_V C_ and ERD_KMI_ reflects the vividness of motor imagery for all tested movements, we plotted the relationships between ERD_sim_ and KMI_total_ or VMI_total_ in **Figure 3**. A significant negative correlation was detected between KMI_total_ and ERD_sim_ between 10% VC and 10% KMI tasks (*r* = -0.688, *p* = 0.001) (**Figure 3A**). As shown in **Figure 3B**, a significant negative correlation was also detected between KMI_total_ score and ERD_sim_ between 40% VC and 40% KMI tasks (*r* = -0.727, *p* < 0.001). However, no significant correlation was detected between VMI_total_ score and ERD_sim_ between 10% VC and 10% KMI tasks (*r* = -0.114, *p* = 0.634) (**Figure 3C**) and no significant correlation was also detected between VMI_total_ score and ERD_sim_ between 40% VC and 40% KMI tasks (*r* = -0.146, *p* = 0.388) (**Figure 3D**).

Similarly, significant negative correlations were detected between KMI_upper_ and ERD_sim_ between 10% VC and 10% KMI tasks (*r* = -0.646, *p* = 0.002), and between KMI_upper_ and ERD_sim_ between 40% VC and 40% KMI tasks (*r* = -0.645, *p* = 0.002). However, no significant correlations were detected between VMI_upper_ and ERD_sim_ between 10% VC and 10% KMI tasks (*r* = -0.221, *p* = 0.348), and between VMI_upper_ and ERD_sim_ between 40% VC and 40% KMI tasks (*r* = -0.218, *p* = 0.356). Comparisons between KVIQ scores and ERD_KMI_ magnitude, and between KVIQ scores and ERD_sim_ are showed in **Table 2**.

**Table 2:** Pearson’s correlation coefficients of measured parameters for all participants.

	KMI_total_	VMI_total_	KMI_upper_	VMI_upper_
ERD_KMI_ 10%	0.487	0.083	0.537	0.191
ERD_KMI_ 40%	0.533	-0.012	0.531	0.084
ERD_sim_ 10%	-**0.688^∗^**	-0.114	-**0.646^∗^**	-0.221
ERD_sim_ 40%	-**0.727^∗^**	-0.146	-**0.645^∗^**	-0.218

*Marked in bold and ^∗^correlations are significant at p < 0.003 (applied Bonferroni adjustment) (N = 20).*

## Discussion

The purpose of the present study was to test the hypothesis that the similarity of changes in EEG sensorimotor rhythm during motor execution and motor imagery related to KMI differ across individuals with different motor imagery ability. We found that the subjective vividness of KMI evaluated by a psychological questionnaire, KVIQ, was associated with the similarity between ERD magnitude during motor execution and that during motor imagery.

### Association Between the Subjective Vividness of Motor Imagery and EEG Sensorimotor Rhythm

A main finding of the present study was that based on Bonferroni-corrected comparisons, the subjective vividness of KMI was not significantly correlated with ERD magnitude itself during motor imagery. The ERD magnitude during motor imagery has been reported to be associated with the motor-evoked potential amplitude in some TMS studies (Hashimoto and Rothwell, 1999; Hummel et al., 2002; Takemi et al., 2013), suggesting that ERD reflects increased neuronal excitability in the corticospinal system. Additionally, as KMI was reported to increase motor-evoked potential amplitudes (Williams et al., 2012), one may assume that the ERD magnitude during motor imagery shows a significant correlation with the subjective vividness of KMI. However, no significant relationship between the ERD magnitude and the KMI_total_ or KMI_upper_ was observed in the present study as previously reported (Vasilyev et al., 2017). This suggests that the subjective vividness of KMI is not associated with how greatly the corticospinal excitability changes from the resting state to the motor imagery state.

However, it is of interest to note that the subjective vividness of KMI was significantly correlated with the similarity between ERD magnitude during motor execution and that during motor imagery. Basically, KMI is performed right after actual motor execution without any feedback in KVIQ. On the other hand, in previous ERD assessments, participants were instructed to perform KMI through the online visual neurofeedback by using ERD as a biomarker, and required to increase ERD magnitude (Shindo et al., 2011; Takemi et al., 2013, 2015). As such, there was a methodological gap in the way of motor imagery between psychological KVIQ tests and neurophysiological ERD assessments. To fill the gap, in the present study, the participants were instructed to perform KMI of the preceding movement with no online visual neurofeedback in ERD assessments, according to the KVIQ procedure. In such a situation, they would use the preceding motor execution as a reference to the KMI. Based on the assumption that motor imagery utilizes common neural substrate with motor execution (Decety, 1996; Jeannerod and Frak, 1999; Gerardin et al., 2000; Hanakawa et al., 2003), it would be reasonable that participants with higher KMI ability are better at changing EEG sensorimotor rhythm during KMI closer to during motor execution. Thus, overall, it is suggested that the subjective vividness of KMI is associated with how we can change the corticospinal excitability during motor imagery similarly to during motor execution.

Our result demonstrated no relationship between vividness of VMI and ERD_sim_. In previous studies, it has been suggested that superior occipital and parietal regions, which are known to be involved in spatial mental imagery and mental navigation even in the absence of any visual input, contribute to VMI of specific body part (Guillot et al., 2009). Moreover, the parallel characteristic between motor execution and motor imagery is reported to be more underlain during KMI than VMI, which is based on the integration of a motor program and corresponding somatosensory feedback (Prinz, 1997; Koch et al., 2004). Thus, it is suggested from these studies that VMI does not accompany with modulation of corticospinal excitability. As the present participants only performed KMI in the physiological experiments, it would be reasonable that the subjective vividness of VMI was not associated with EEG sensorimotor rhythm.

We found no significant difference in ERD_KMI_ between 10% KMI and 40% KMI tasks. There are several studies investigating the association between imagined muscle contraction strength and corticospinal excitability (Park and Li, 2011; Mizuguchi et al., 2013; Helm et al., 2015). In some of them, significant differences in motor-evoked potential amplitudes during KMI were observed in between low- and high-imagined contraction strength (i.e., 10 vs. 60% of maximal effort), suggesting difference in the corticospinal excitability depending on imagined contraction strength (Mizuguchi et al., 2013; Helm et al., 2015). Our result was inconsistent with these studies. This could be due to the little contrast between two strengths we set in the experiment to make a significant difference in corticospinal excitability. In the present study, % of MVC of higher strength was set lower than the previous studies to prevent muscle fatigue, because participants were required to repeat many VC and KMI tasks according to KVIQ procedure. Additionally, we have found that there was no significant difference in ERD_V C_ between 10% VC task and 40% VC task. This might lead to little difference in reference used for KMI between 10% VC task and 40% VC task. Because of these reasons, difference in ERD magnitude between two strengths would be smaller than we expected. Although no difference in an effect of strength was observed, significant difference in an effect of task was detected. No significant difference between motor imagery and motor execution in arrival time of ERD peak in alpha band was observed, but ERD magnitude during motor execution is significantly larger than the one during motor imagery (Duann and Chiou, 2016). Additionally, in fMRI studies (Gerardin et al., 2000; Zich et al., 2015), similar activation maps during motor imagery and motor execution were shown. However, in the activation map during motor execution, the activation was more connected and solid, and its area was larger than the one during motor imagery. The generating process of motor execution is similar to the one of motor imagery, but motor execution is different from motor imagery in that any overt motor output or EMG is observed. Although motor execution needs motor command and motor unit to induce muscle contraction, motor imagery is mental rehearsal that does not accompany with motor output. Therefore, we considered that detecting the significant difference between ERD magnitude during motor imagery and the one during motor execution is reasonable result.

### Technical Limitations of the Present Study

In the present study, scalp EEGs were obtained only from five positions over the sensorimotor cortex dominating right upper limb. One might claim that recorded EEG included not only the sensorimotor rhythm but also other rhythms such as the occipital rhythm. During relaxed wakefulness, the human brain exhibits pronounced rhythmic electrical activity in the alpha band. This activity consists of three main components (Hughes and Crunelli, 2005): the classic occipital alpha rhythm related to visual function (Scheeringa et al., 2011), the Rolandic mu rhythm related to somatosensory function (Arroyo et al., 1993), and the midtemporal third rhythm which can be recorded by scalp EEGs from ones with bone defects (Neidermeyer, 1991). To extract the unique EEG from the sensorimotor area, we applied the Laplacian filter to the raw EEGs in the present analyses. In addition, our results demonstrated no significant correlations between VMI scores and ERD_sim_. Thus, although we cannot totally deny the possibility that recorded EEG included several rhythms other than the sensorimotor rhythm, its effect would be limited.

In the present physiological experiments, the tasks performed by the participants were just the repetition of VCs and KMIs of wrist extension. Thus, we cannot judge whether the association between KMI ability and EEG sensorimotor rhythm can be obtained even when we perform similar physiological experiments for different muscles and/or different motor tasks. Indeed, some previous TMS studies have demonstrated muscle- and/or movement-specific effects of motor imagery on the corticospinal excitability (Hashimoto and Rothwell, 1999; Li et al., 2005; Stinear et al., 2006). In addition, it leaves much room for discussion about whether the neural substrates determining the motor imagery ability and the ones modulating corticospinal excitability are common or not, and if not, how they influence one another directly or indirectly. Besides the neural substrates, it is reported a significant increase of heart rate and respiration rate during motor imagery (Decety et al., 1993; Oishi et al., 2000). Therefore, individual differences relate to motor imagery ability may be detected for other physiological indices such as heart rate and respiration rate. Further investigation would be needed regarding the generality of association between the subjective vividness of motor imagery and EEG sensorimotor rhythm across body parts, movements used for motor imagery, and individuals.

### Application for Motor Imagery Training

Recently, several studies have reported positive effects of the brain–machine interface rehabilitation for hand paralysis following stroke (Müller et al., 2003; Shindo et al., 2011; Ang et al., 2014). In these studies, ERD was used as a biomarker and the patients were required to increase ERD during motor imagery through the online visual neurofeedback. More importantly, for patients, “motor imagery” is the task to try imagining their paretic body-part with severely limited range of motion. Thus, it is reasonable that neurorehabilitation aiming at increment of corticospinal excitability induced neural plasticity and improvement in motor function. On the other hand, “motor imagery” for healthy people is totally different from the one performed by patients because they can easily increase corticospinal excitability enough to move their intending body-part voluntarily. For healthy people, “motor imagery” would be the task to picture their own movements in their mind by referring their own bodily movements. Therefore, in terms of motor imagery training for healthy humans, for example, athletes, neurofeedback system just to enhance corticospinal excitability would not be able to make their imagery more vivid. Rather, it may be important for healthy humans to train their ability to modulate the sensorimotor rhythm during motor imagery closer to during motor execution. To examine such effects of motor imagery trainings, interpretations of the causality between the psychological and neurophysiological aspects of motor imagery would be facilitated.

## Conclusion

The present study demonstrated that the subjective vividness of KMI was significantly associated with the similarity between ERD magnitude during motor execution and that during motor imagery. The data suggest that in healthy individuals with higher motor imagery ability from a first-person perspective, KMI efficiently engages the shared cortical circuits corresponding with motor execution, including the sensorimotor cortex, with high compliance.

## Data Availability Statement

The raw data supporting the conclusions of this manuscript will be made available by the authors, without undue reservation, to any qualified researcher.

## Author Contributions

HT, JUb, and JUy designed the work and wrote the paper. HT acquired and analyzed the data.

## Conflict of Interest Statement

The authors declare that the research was conducted in the absence of any commercial or financial relationships that could be construed as a potential conflict of interest.

## References

[B1] Aftanas L. I. Varlamov A. A. Pavlov S. V. Makhnev V. P. Reva N. V. (2002). Time-dependent cortical asymmetries induced by emotional arousal: EEG analysis of event-related synchronization and desynchronization in individually defined frequency bands. *Int. J. Psychophysiol.* 44 67–82. 10.1016/S0167-8760(01)00194-5 11852158

[B2] Ang K. K. Guan C. Phua K. S. Wang C. Zhou L. Tang K. Y. (2014). Brain-computer interface-based robotic end effector system for wrist and hand rehabilitation: results of a three-armed randomized controlled trial for chronic stroke. *Front. Neuroeng.* 7:30. 10.3389/fneng.2014.00030 25120465 PMC4114185

[B3] Arroyo S. Lesser R. P. Gordon B. Uematsu S. Jackson D. Webber R. (1993). Functional significance of the mu rhythm of human cortex: an electrophysiologic study with subdural electrodes. *Electroencephalogr. Clin. Neurophysiol.* 87 76–87. 10.1016/0013-4694(93)90114-B 7691544

[B4] Blankertz B. Sannelli C. Halder S. Hammer E. M. Kubler A. Muller K. R. (2010). Neurophysiological predictor of SMR-based BCI performance. *Neuroimage* 51 1303–1309. 10.1016/j.neuroimage.2010.03.022 20303409

[B5] Cassim F. Szurhaj W. Sediri H. Devos D. Bourriez J.-L. Poirot I. (2000). Brief and sustained movements: differences in event-related (de)synchronization (ERD/ERS) patterns. *Clin. Neurophysiol.* 111 2032–2039. 10.1016/S1388-2457(00)00455-7 11068239

[B6] Decety J. (1996). Do imagined and executed actions share the same neural substrate? *Brain Res. Cogn. Brain Res.* 3 87–93. 10.1016/0926-6410(95)00033-X 8713549

[B7] Decety J. Grezes J. (1999). Neural mechanisms subserving the perception of human actions. *Trends Cogn. Sci.* 3 172–178. 10.1016/S1364-6613(99)01312-1 10322473

[B8] Decety J. Jeannerod M. Durozard D. Baverel G. (1993). Central activation of autonomic effectors during mental simulation of motor actions in man. *J. Physiol.* 461 549–563. 10.1113/jphysiol.1993.sp019528 8102402 PMC1175272

[B9] Duann J. R. Chiou J. C. (2016). A comparison of independent event-related desynchronization responses in motor-related brain areas to movement execution, movement imagery, and movement observation. *PLoS One* 11:e0162546. 10.1371/journal.pone.0162546 27636359 PMC5026344

[B10] Formaggio E. Storti S. F. Avesani M. Cerini R. Milanese F. Gasparini A. (2008). EEG and FMRI coregistration to investigate the cortical oscillatory activities during finger movement. *Brain Topogr.* 21 100–111. 10.1007/s10548-008-0058-1 18648924

[B11] Formaggio E. Storti S. F. Cerini R. Fiaschi A. Manganotti P. (2010). Brain oscillatory activity during motor imagery in EEG-fMRI coregistration. *Magn. Reson. Imaging* 28 1403–1412. 10.1016/j.mri.2010.06.030 20850237

[B12] Gerardin E. Sirigu A. Lehéricy S. Poline J.-B. Gaymard B. Marsault C. (2000). Partially overlapping neural networks for real and imagined hand movements. *Cereb. Cortex* 10 1093–1104. 10.1093/cercor/10.11.1093 11053230

[B13] Guillot A. Collet C. Nguyen V. A. Malouin F. Richards C. Doyon J. (2009). Brain activity during visual versus kinesthetic imagery: an fMRI study. *Hum. Brain Mapp.* 30 2157–2172. 10.1002/hbm.20658 18819106 PMC6870928

[B14] Hall C. R. Pongrac J. Buckholz E. (1985). The measurement of imagery ability. *Hum. Mov. Sci.* 4 107–118. 10.1016/0167-9457(85)90006-5

[B15] Hanakawa T. Immisch I. Toma K. Dimyan M. A. Gelderen P. V. Hallett M. (2003). Functional properties of brain areas associated with motor execution and imagery. *J. Neurophysiol.* 89 989–1002. 10.1152/jn.00132.2002 12574475

[B16] Hashimoto R. Rothwell J. C. (1999). Dynamic changes in corticospinal excitability during motor imagery. *Exp. Brain Res.* 125 75–81. 10.1007/s002210050660 10100979

[B17] Hashimoto Y. Ushiba J. Kimura A. Liu M. Tomita Y. (2010). Correlation between EEG-EMG coherence during isometric contraction and its imaginary execution. *Acta Neurobiol. Exp.* 70 76–85. 20407489 10.55782/ane-2010-1776

[B18] Helm F. Marinovic W. Krüger B. Munzert J. Riek S. (2015). Corticospinal excitability during imagined and observed dynamic force production tasks: effortfulness matters. *Neuroscience* 290 398–405. 10.1016/j.neuroscience.2015.01.050 25639231

[B19] Hetu S. Gregoire M. Saimpont A. Coll M. P. Eugene F. Michon P. E. (2013). The neural network of motor imagery: an ALE meta-analysis. *Neurosci. Biobehav. Rev.* 37 930–949. 10.1016/j.neubiorev.2013.03.017 23583615

[B20] Hughes S. W. Crunelli V. (2005). Thalamic mechanisms of EEG alpha rhythms and their pathological implications. *Neuroscientist* 11 357–372. 10.1177/1073858405277450 16061522

[B21] Hummel F. Andres F. Altenmüller E. Dichgans J. Gerloff C. (2002). Inhibitory control of acquired motor programmes in the human brain. *Brain* 125 404–420. 10.1093/brain/awf030 11844740

[B22] Hummel F. Saur R. Lasogga S. Plewnia C. Erb M. Wildgruber D. (2004). To act or not to act. Neural correlates of executive control of learned motor behavior. *Neuroimage* 23 1391–1401. 10.1016/j.neuroimage.2004.07.070 15589103

[B23] Jeannerod M. Frak V. (1999). Mental imaging of motor activity in humans. *Curr. Opin. Neurobiol.* 9 735–739. 10.1016/S0959-4388(99)00038-0 10607647

[B24] Kaplan A. Vasilyev A. Liburkina S. Yakovlev L. (2016). “Poor BCI performers still could benefit from motor imagery training,” in *Proceedings of the 10th International Conference on Foundations of Augmented Cognition: Neuroergonomics and Operational Neuroscience* Vol. 9743 Part I, (New York, NY: Springer-Verlag New York, Inc.), 46–56. 10.1007/978-3-319-39955-3_5

[B25] Koch I. Keller P. Prinz W. (2004). The Ideomotor approach to action control: implications for skilled performance. *Int. J. Sport Exerc. Psychol.* 2 362–375. 10.1080/1612197X.2004.9671751

[B26] Li S. Stevens J. A. Kamper D. G. Rymer W. Z. (2005). The movement-specific effect of motor imagery on the premotor time. *Mot. Control* 9 119–128. 10.1123/mcj.9.2.119 15995254

[B27] Lorey B. Pilgramm S. Bischoff M. Stark R. Vaitl D. Kindermann S. (2011). Activation of the parieto-premotor network is associated with vivid motor imagery–a parametric FMRI study. *PLoS One* 6:e20368. 10.1371/journal.pone.0020368 21655298 PMC3105023

[B28] Malouin F. Richards C. L. Durand A. (2010). Normal aging and motor imagery vividness: implications for mental practice training in rehabilitation. *Arch. Phys. Med. Rehabil.* 91 1122–1127. 10.1016/j.apmr.2010.03.007 20537312

[B29] Malouin F. Richards C. L. Jackson P. L. Lafleur M. F. Durand A. Doyon J. (2007). The kinesthetic and visual imagery questionnaire (kviq) for assessing motor imagery in persons with physical disabilities: a reliability and construct validity study. *J. Neurol. Phys. Ther.* 31 20–29. 10.1097/01.npt.0000260567.24122.64 17419886

[B30] Marchesotti S. Bassolino M. Serino A. Bleuler H. Blanke O. (2016). Quantifying the role of motor imagery in brain-machine interfaces. *Sci. Rep.* 6:24076. 10.1038/srep24076 27052520 PMC4823701

[B31] McFarland D. J. McCane L. M. David S. V. Wolpaw J. R. (1997). Spatial filter selection for EEG-based communication. *Electroencephalogr. Clin. Neurophysiol.* 103 386–394. 10.1016/S0013-4694(97)00022-2 9305287

[B32] Mizuguchi N. Umehara I. Nakata H. Kanosue K. (2013). Modulation of corticospinal excitability dependent upon imagined force level. *Exp. Brain Res.* 230 243–249. 10.1007/s00221-013-3649-3 23877227

[B33] Mokienko O. A. Chervyakov A. V. Kulikova S. N. Bobrov P. D. Chernikova L. A. Frolov A. A. (2013). Increased motor cortex excitability during motor imagery in brain-computer interface trained subjects. *Front. Comput. Neurosci.* 7:168. 10.3389/fncom.2013.00168 24319425 PMC3837244

[B34] Müller G. R. Neuper C. Rupp R. Keinrath C. Gerner H. J. Pfurtscheller G. (2003). Event-related beta EEG changes during wrist movements induced by functional electrical stimulation of forearm muscles in man. *Neurosci. Lett.* 340 143–147. 10.1016/S0304-3940(03)00019-3 12668257

[B35] Muller-Putz G. R. Scherer R. Brauneis C. Pfurtscheller G. (2005). Steady-state visual evoked potential (SSVEP)-based communication: impact of harmonic frequency components. *J. Neural. Eng.* 2 123–130. 10.1088/1741-2560/2/4/008 16317236

[B36] Nagamori S. Tanaka H. (2016). ERD analysis method in motor imagery brain–computer interfaces for accurate switch input. *Artif. Life Robot.* 22 83–89. 10.1007/s10015-016-0336-z

[B37] Neidermeyer E. (1991). The “third rhythm”: further observations. *Clin. Electroencephalogr.* 22 83–96. 10.1177/155005949102200208 2032348

[B38] Neuper C. Wörtz M. Pfurtscheller G. (2006). ERD/ERS patterns reflecting sensorimotor activation and deactivation. *Prog. Brain Res.* 159 211–222. 10.1016/S0079-6123(06)59014-4 17071233

[B39] Nunez P. L. Srinivasan R. (2006). A theoretical basis for standing and traveling brain waves measured with human EEG with implications for an integrated consciousness. *Clin. Neurophysiol.* 117 2424–2435. 10.1016/j.clinph.2006.06.754 16996303 PMC1991284

[B40] Oishi K. Kasai T. Maeshima T. (2000). Autonomic response specificity during motor imagery. *J. Physiol. Anthropol. Appl. Hum. Sci.* 19 255–261. 10.2114/jpa.19.255 11204872

[B41] Oostra K. M. Oomen A. Vanderstraeten G. Vingerhoets G. (2015). Influence of motor imagery training on gait rehabilitation in sub-acute stroke: a randomized controlled trial. *J. Rehabil. Med.* 47 204–209. 10.2340/16501977-1908 25403275

[B42] Oostra K. M. Van Bladel A. Vanhoonacker A. C. Vingerhoets G. (2016). Damage to fronto-parietal networks impairs motor imagery ability after stroke: a voxel-based lesion symptom mapping study. *Front. Behav. Neurosci.* 10:5. 10.3389/fnbeh.2016.00005 26869894 PMC4740776

[B43] Park W. H. Li S. (2011). No graded responses of finger muscles to TMS during motor imagery of isometric finger forces. *Neurosci. Lett.* 494 255–259. 10.1016/j.neulet.2011.03.027 21406217 PMC3085454

[B44] Pfurtscheller G. (1981). Central beta rhythm during sensorimotor activities in man. *Electroencephalogr. Clin. Neurophysiol.* 51 253–264. 10.1016/0013-4694(81)90139-5 6163614

[B45] Pfurtscheller G. (1992). Event-related synchronization (ERS): an electrophysiological correlate of cortical areas at rest. *Electroencephalogr. Clin. Neurophysiol.* 83 62–69. 10.1016/0013-4694(92)90133-3 1376667

[B46] Pfurtscheller G. Aranibar A. (1979). Evaluation of event-related desynchronization (ERD) preceding and following voluntary self-paced movement. *Electroencephalogr. Clin. Neurophysiol.* 46 138–146. 10.1016/0013-4694(79)90063-4 86421

[B47] Pfurtscheller G. Berghold A. (1989). Patterns of cortical activation during planning of voluntary movement. *Electroencephalogr. Clin. Neurophysiol.* 72 250–258. 10.1016/0013-4694(89)90250-2 2465128

[B48] Pfurtscheller G. Brunner C. Schlogl A. Lopes da Silva F. H. (2006). Mu rhythm (de)synchronization and EEG single-trial classification of different motor imagery tasks. *Neuroimage* 31 153–159. 10.1016/j.neuroimage.2005.12.003 16443377

[B49] Pfurtscheller G. Lopes da Silva F. H. (1999). Event-related EEG/MEG synchronization and desynchronization: basic principles. *Clin. Neurophysiol.* 110 1842–1857. 10.1016/S1388-2457(99)00141-8 10576479

[B50] Pfurtscheller G. Neuper C. (2006). Future prospects of ERD/ERS in the context of brain–computer interface (BCI) developments. *Prog. Brain Res.* 159 433–437. 10.1016/S0079-6123(06)59028-4 17071247

[B51] Prinz W. (1997). Perception and action planning. *Eur. J. Cogn. Psychol.* 9 129–154. 10.1080/713752551

[B52] Scheeringa R. Mazaheri A. Bojak I. Norris D. G. Kleinschmidt A. (2011). Modulation of visually evoked cortical FMRI responses by phase of ongoing occipital alpha oscillations. *J. Neurosci.* 31 3813–3820. 10.1523/JNEUROSCI.4697-10.2011 21389236 PMC6622780

[B53] Shindo K. Kawashima K. Ushiba J. Ota N. Ito M. Ota T. (2011). Effects of neurofeedback training with an electroencephalogram-based brain-computer interface for hand paralysis in patients with chronic stroke: a preliminary case series study. *J. Rehabil. Med.* 43 951–957. 10.2340/16501977-0859 21947184

[B54] Stinear C. M. Byblow W. D. Steyvers M. Levin O. Swinnen S. P. (2006). Kinesthetic, but not visual, motor imagery modulates corticomotor excitability. *Exp. Brain Res.* 168 157–164. 10.1007/s00221-005-0078-y 16078024

[B55] Takemi M. Masakado Y. Liu M. Ushiba J. (2013). Event-related desynchronization reflects downregulation of intracortical inhibition in human primary motor cortex. *J. Neurophysiol.* 110 1158–1166. 10.1152/jn.01092.2012 23761697

[B56] Takemi M. Masakado Y. Liu M. Ushiba J. (2015). Sensorimotor event-related desynchronization represents the excitability of human spinal motoneurons. *Neuroscience* 297 58–67. 10.1016/j.neuroscience.2015.03.045 25839147

[B57] Tsuchimoto S. Shibusawa S. Mizuguchi N. Kato K. Ebata H. Liu M. (2017). Resting-state fluctuations of eeg sensorimotor rhythm reflect bold activities in the pericentral areas: a simultaneous EEG-fMRI study. *Front. Hum. Neurosci.* 11:356. 10.3389/fnhum.2017.00356 28729830 PMC5498521

[B58] Vasilyev A. Liburkina S. Yakovlev L. Perepelkina O. Kaplan A. (2017). Assessing motor imagery in brain-computer interface training: psychological and neurophysiological correlates. *Neuropsychologia* 97 56–65. 10.1016/j.neuropsychologia.2017.02.005 28167121

[B59] Vuckovic A. Osuagwu B. A. (2013). Using a motor imagery questionnaire to estimate the performance of a brain-computer interface based on object oriented motor imagery. *Clin. Neurophysiol.* 124 1586–1595. 10.1016/j.clinph.2013.02.016 23535455

[B60] Williams J. Pearce A. J. Loporto M. Morris T. Holmes P. S. (2012). The relationship between corticospinal excitability during motor imagery and motor imagery ability. *Behav. Brain Res.* 226 369–375. 10.1016/j.bbr.2011.09.014 21939692

[B61] Yuan H. Liu T. Szarkowski R. Rios C. Ashe J. He B. (2010). Negative covariation between task-related responses in alpha/beta-band activity and BOLD in human sensorimotor cortex: an EEG and fMRI study of motor imagery and movements. *Neuroimage* 49 2596–2606. 10.1016/j.neuroimage.2009.10.028 19850134 PMC2818527

[B62] Zich C. Debener S. Kranczioch C. Bleichner M. G. Gutberlet I. De Vos M. (2015). Real-time EEG feedback during simultaneous EEG-fMRI identifies the cortical signature of motor imagery. *Neuroimage* 114 438–447. 10.1016/j.neuroimage.2015.04.020 25887263

