# Supplementary material for: Subjective Vividness of Kinesthetic Motor Imagery Is Associated With the Similarity in Magnitude of Sensorimotor Event-Related Desynchronization Between Motor Execution and Motor Imagery
Source: Front Hum Neurosci. 2018 Jul 31;12:295. doi: 10.3389/fnhum.2018.00295 (PMC6079198; doi:10.3389/fnhum.2018.00295)
Supplement: Supplementary file 1 [file Image_1.pdf]

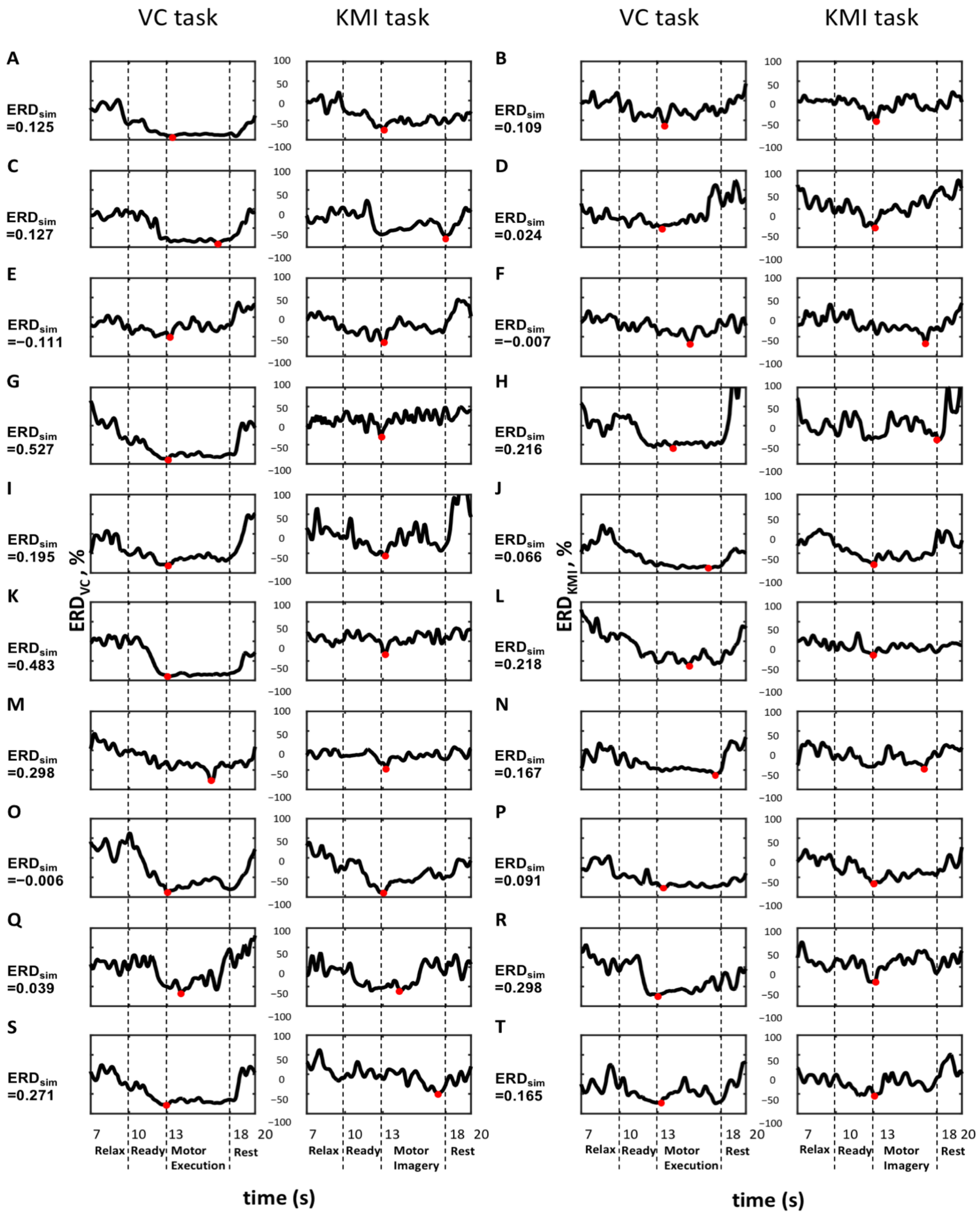

Supplementary Figure 1. ERD curves for all participants. A-T corresponds to participant A-T. The left side of each participant's figure is the ERD of VC task and the right side shows the ERD of KMI task. It also shows ERD_{sim} for all participants.
